# Ceramic bone graft substitute (Mg-HA) in spinal fusion: A prospective pilot study

**DOI:** 10.3389/fbioe.2022.1050495

**Published:** 2022-11-17

**Authors:** Cristiana Griffoni, Giuseppe Tedesco, Valentina Canella, Angelo Nataloni, Alberto Zerbi, Giovanni Tosini, Alessandro Gasbarrini, Giovanni Barbanti-Brodano

**Affiliations:** ^1^ Department of Spine Surgery, IRCCS Istituto Ortopedico Rizzoli, Bologna, Italy; ^2^ Finceramica Faenza S.p.A., Faenza, Italy; ^3^ Istituti Clinici Iseni, Fondazione Iseni y Nervi, Lonate Pozzolo, Italy

**Keywords:** lumbar degenerative disc disease, spinal fusion, bone graft substitute, Brantigan score, bioceramics

## Abstract

**Background:** Iliac crest bone graft (ICBG) is considered the gold standard for spine surgical procedures to achieve a successful fusion due to its known osteoinductive and osteoconductive properties. However, complications related to harvesting procedure and donor site morbidity have been largely reported in the literature, favoring the development of a wide range of alternative products to be used as bone graft extenders or substitutes for spine fusion. Among all, ceramic-based biomaterials have been widely studied and employed in the last years as bone graft substitutes.

**Methods:** We report here the results of a prospective pilot study aimed to evaluating the grade of ossification obtained by the use of an Mg-doped hydroxyapatite (HA) product to achieve postero-lateral fusion in degenerative spine diseases.

**Results:** Results show a successful degree of fusion of about 62% at the 12-month follow-up and an improvement of quality of life and health status following surgery, as evaluated by clinical scores (ODI, VAS, and EQ-5L). No adverse events related to the material were reported.

**Conclusion:** The present pilot study shows the effectiveness and the safety profile of an Mg-doped HA bone graft substitute used to achieve postero-lateral fusion in the treatment of degenerative spine diseases, laying down the basis for further larger clinical investigations.

## Introduction

During the past few decades, spinal fusion procedures have significantly increased to treat a wide range of spinal disorders of degenerative, traumatic, and oncological origin (Reisener et al., 2020). Autologous bone graft from the iliac crest (ICBG) has been classically used to provide spinal fusion and immediate structural support and is still considered the “gold standard” because of the osteoconductive and osteoinductive properties, which allow excellent fusion rates (Banwart et al., 1995; Arrington et al., 1996; Ahlmann et al., 2002; Dimar et al., 2009; Kim et al., 2009). Nevertheless, autologous bone harvesting has shown limitations and significant drawbacks, including superficial infection, wound complications, sensory abnormalities, persistent pain, and hematomas, as well as need for reoperation, scarring, and graft site fracture (Dimar et al., 2009; Kim et al., 2009; Miyazaki et al., 2009; Gruskay et al., 2014).

To overcome all these limitations, different alternatives have been developed, clinically tested, and currently available on the market, including allograft (Park et al., 2013; Gupta et al., 2015), bone morphogenetic protein, demineralized bone matrix (DBM) (Abdullah et al., 2011; Hsu et al., 2012; Fischer et al., 2013), BMPs (Carragee et al., 2011; Kannan et al., 2015), mesenchymal stem cells (MSCs), and bioceramics (Fischer et al., 2013).

Although extensively studied, clinical data available for all these materials are often heterogeneous in quality, type of study and evaluations performed, and conclusions reached (Miyazaki et al., 2009; Abdullah et al., 2011; Alsaleh et al., 2012; Hsu et al., 2012).

Bioactive ceramics (i.e., tricalcium phosphate, calcium phosphate, calcium sulfate, hydroxyapatite, and collagen) (Nickoli and Hsu, 2014) are synthetic products which have been developed as osteoconductive scaffolds with chemico-physical properties very similar to the mineral component of human bone (Korovessis et al., 2005; Alsaleh et al., 2012; Gao et al., 2014; Kaiser et al., 2014). These biomaterials are able to stimulate cell proliferation and differentiation and bone tissue regeneration/remodeling while undergoing in the meanwhile slow resorption. Among all, HA is the most similar, for the chemico-physical composition and stoichiometric formula (Ca/P ratio = 1.67), to the mineralized phase of human bone.

To further improve their features, new generation HA-based biomaterials have been developed with superior properties, strongly influenced by the nature of components, the composition, and the morphology. Calcium ions, phosphate ions, and hydroxyl groups can be replaced by other ions, and studies on animal models have demonstrated that HA-substituted ions enable the crystal cell structure of ceramic derivatives to become unstable and more biologically active, thereby promoting rapid cell-mediated material resorption, new bone formation, and remodeling (Nandi et al., 2010).

We previously reported the results of a pre-clinical study performed using an HA-doped bioceramic (SintLife, Finceramica, Faenza, S.p.A., Italy) enriched in magnesium (Mg) to induce spinal fusion in an animal model (Barbanti Bròdano et al., 2014).

In this study, we report the results of a prospective clinical study performed using the same Mg-substituted HA bone graft in a cohort of patients undergoing postero-lateral fusion for degenerative lumbar spine diseases.

The primary aim of the study was the evaluation of the degree of fusion and new bone formation achieved by the use of the bone graft substitute SintLife. The secondary aim was the assessment of the patients’ state of health by the evaluation of clinical scores (Oswestry Disability Index, visual analog scale, and EuroQol (EQ-5D)) in the post-operative period. The third aim was to study the SintLife safety profile in human subjects.

## Materials and methods

### Clinical study design

A prospective pilot clinical study was conducted at our center from February 2017 to March 2020, following the approval of the Local Ethics Committee (protocol number 0001112: “Use of the bone substitute SintLife in spinal surgical procedures. A pilot study”). The study was performed in line with the principles of the Declaration of Helsinki. The study design was a pilot study because the bone graft substitute was not used routinely in our center for spinal surgery, and the pilot study allowed evaluating the results obtained in a small scale, providing the basis for larger investigations. Consequently, the sample size was calculated according to the standard clinical activities ordinarily carried out by the center involved in the study and by the consideration reported in the following paragraph, for which the total number of 20 subjects involved was considered sufficient for the assessment of the variables under analysis. This is not a comparative study, and comparison shall be performed only with literature data.

Consecutive patients who had indications of single- or multi-level postero-lateral spinal fusion due to degenerative lumbar spine diseases were screened to be included in the study, after providing written informed consent. The enrolment period was from February 2017 to September 2019, and patients were followed up for 18 months, with the exception of three patients who dropped out because of adverse events occurred after surgery.

Specific inclusion criteria were as follows: skeletally mature subjects, at least 18 years of age at the time of surgery with symptomatic spinal degenerative disc disease requiring postero-lateral fusion at the L1–S1 tract and patients participating in the study who provided informed signed consent. Patient exclusion criteria were applied in the case of local or systemic infections, inflammatory or autoimmune disease, hypercalcemia, coagulation/metabolic disorders, insulin-dependent diabetes, allergic to calcium phosphate salts, drugs, or medical devices, tumor pathologies, alcohol or drug abuse, pregnancy, pharmacologic therapies which might have influenced bone regeneration processes (i.e., chemotherapeutic drugs), and revision surgery.

### Surgical procedure

A conventional posterior approach for lumbar spinal fusion was performed. After the positioning of pedicle screws, decompression of the cauda and nerve roots was achieved with hemilaminectomy and foraminotomy. SintLife was apposed on the hemi-laminae and transverse process on the contralateral side of the hemilaminectomy. On the hemilaminectomy side, foramino-arthrectomy was performed to insert the interbody fusion cage; therefore, on that side, the dura and the nerve roots were exposed, and SintLife could not be put there.

### Biomaterial

SintLife is a CE-marked, implantable, non-active bone graft substitute, available in the form of a paste, composed of biomimetic hydroxyapatite (HA) enriched in magnesium ions (Mg-HA) in a similar amount as in the one found in human bones. Mg^2+^ ions are introduced into the HA crystalline cell in the same position and percentage found in the mineral phase of human bone. Previous studies have been demonstrated that the presence of Mg^2+^ ions deform the structure of the crystalline cell of HA, making it unstable and biologically active, thus favoring new bone formation, bone remodeling, and rapid cell-mediated resorption of the material. Furthermore, Mg-HA actively interacts with water molecules to rapidly capture key proteins involved in osteogenesis. The specific chemical and biomimetic composition, structured geometry, and surface properties allow SintLife to be remodeled and reabsorbed by osteoprogenitor cells in a physiologically adequate period (6–18 months) to promote quick and effective physiological bone regeneration. During the remodeling phase, osteoclast reabsorption activity has been observed around the bone graft material, up to the complete regeneration of new bone (Barbanti Bròdano et al., 2014).

### Result assessment

Radiographic and clinical data were collected before surgery and at 6- and 12–18-month follow-ups. Radiographic images were used to determine the degree of fusion and bone regeneration. Clinical scores such as the Oswestry Disability Index (ODI), the visual analog scale (VAS), and the EuroQoL-5L (EQ-5L) were used to evaluate the improvement of the patient’s health quality following surgery.

Bone regeneration and the degree of fusion were determined by an independent radiologist on CT scan analysis. Bone regeneration was identified as the presence of a continuous trabecular bone bridge together with the lack of radiolucency as assessed by diagnostic imaging (CT scan), and it was evaluated by Brantigan classification (Table 1), which assesses the spinal fusion from grade A (pseudoarthrosis) to grade E (certain fusion).

**TABLE 1:** Brantigan classification of spinal fusion.

Classification	Description
A- Obvious radiographic pseudoarthrosis	Pseudoarthrosis, collapse of construct, loss of disc height, vertebral slip, broken screw, displacement of the cage, and resorption of the bone graft
B- Probable pseudoarthrosis	Significant resorption of the bone graft and major lucency or gap visible in the fusion area >2 mm
C- Radiographic status uncertain	A small lucency or gap may be visible with at least half of the graft area showing no lucency between the graft bone and the vertebral bone
D- Probable radiographic fusion	Bone bridges the entire fusion area with at least the density originally achieved at surgery. There should be no lucency between the graft bone and the vertebral bone
E- Radiographic fusion	The bone in the fusion area is denser and more mature than originally achieved at surgery; there is no interface between the donor bone and the vertebral bone: a sclerotic line between the graft bone and the vertebral bone indicates solid fusion. Other indicators of solid fusion are fusion at facet joints and anterior progression of the graft in the disc.

Clinical evaluations were collected before surgery and at follow-up visits using the following scores: the Oswestry Disability Index (ODI) for the quantification of patient’s disability for low back pain, the visual analog scale (VAS) for patient’s pain intensity, and EuroQol (EQ-5D) for measurement of the quality of life. The incidence of adverse events, complications, unattended reactions, and incidents was recorded.

### Statistical analysis

No sample size calculation was performed because of the study design (i.e., pilot study). Considering the small number of patients treated and the heterogeneity of the patient population included in the study, no specific statistical analyses have been carried out. Descriptive statistical analysis has been provided for clinical scores (VAS, ODI, and EQ-5D).

Results are presented as the number (n), mean ± standard deviation, and percentage, as appropriate. Changes from the baseline to follow-up scores were analyzed using Student’s t-test. The level of statistical significance was set at *p* < 0.05. SAS software 9.201 was used. Assessment of fusion and the incidence and type of any adverse event recorded have been reported.

## Results

**FIGURE 1:**
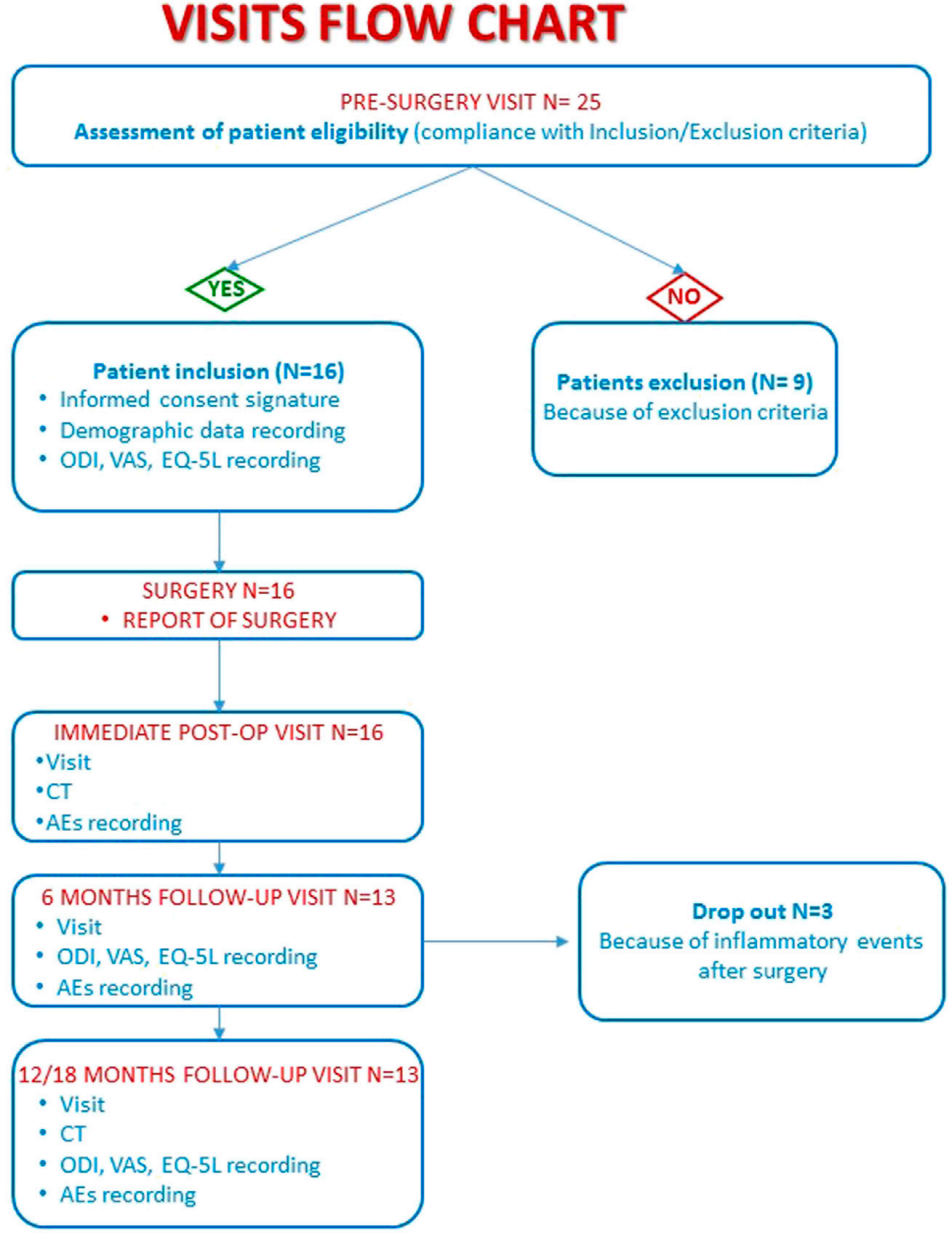
Flow chart of pre-operative and post-operative visits.

Because of enrolment delays due to the COVID-19 pandemic, the expected sample size of 20 subjects was not reachable, and a final number of 16 subjects were enrolled for the study. The flow chart of the study is represented in Figure 1. In this figure, the number of patients included in the analysis at each time point and the reason for dropout are reported. All the enrolled patients signed the written informed consent for the study and underwent postero-lateral fusion for degenerative lumbar spine diseases.

As reported in Table 2, there were seven males (44%) and nine females (56%), with a mean age of about 54.2 years (age range: 34–80); 37.5% of subjects were aged between 55 and 64 years; 31.3% (5) were aged between 35 and 44 years. The majority of patients were treated for spondylolisthesis (43.9%) and degenerative disc disease (37.5%). An interbody cage was implanted in 13/16 patients through a transforaminal lumbar interbody fusion (TLIF) approach on the side of the hemilaminectomy.

**TABLE 2:** Demographic data.

	Number of patients	%
Gender		
Male	7	43.8
Female	9	56.3
Age at surgery		
25–34 years	1	6.3
35–44 years	5	31.3
45–54 years	1	6.3
55–64 years	6	37.5
65–74 years	1	6.3
≥ 75 years	2	12.5
Etiology		
Spondylolisthesis	7	43.9
Degenerative disc disease	6	37.5
Scoliosis	1	6.2
Lumbar stenosis	1	6.2
Kyphosis	1	6.2
Levels treated		
1 level	9 (9 levels)	56
2 levels	3 (6 levels)	19
3 levels	4 (12 levels)	25

**FIGURE 2:**
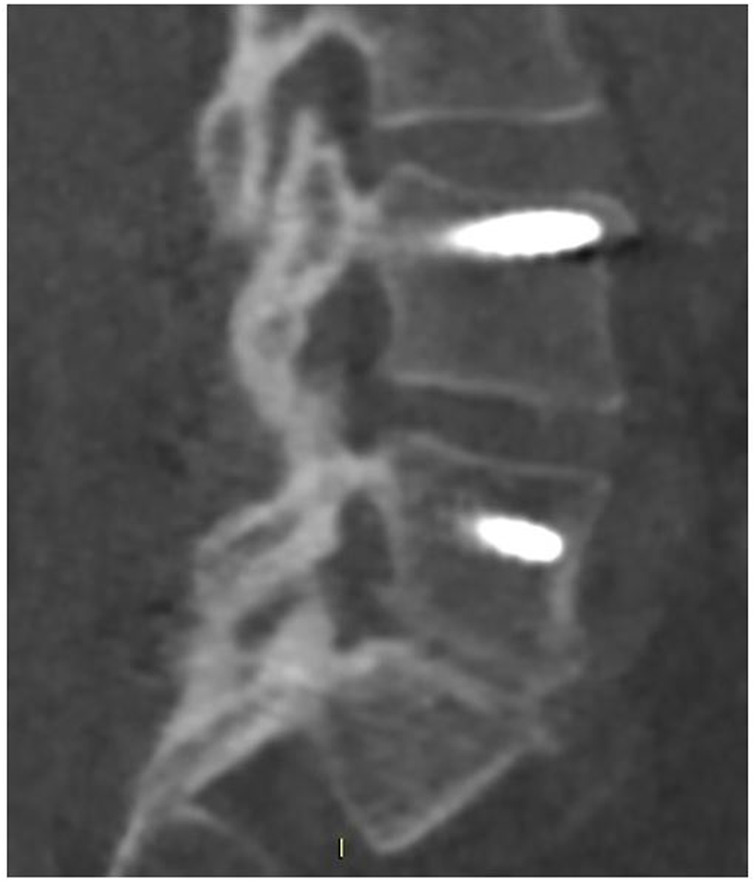
CT scan performed at 18-month follow-up, showing radiographic fusion at levels L4–L5 and L5–S1 where SintLife was used.

**FIGURE 3:**
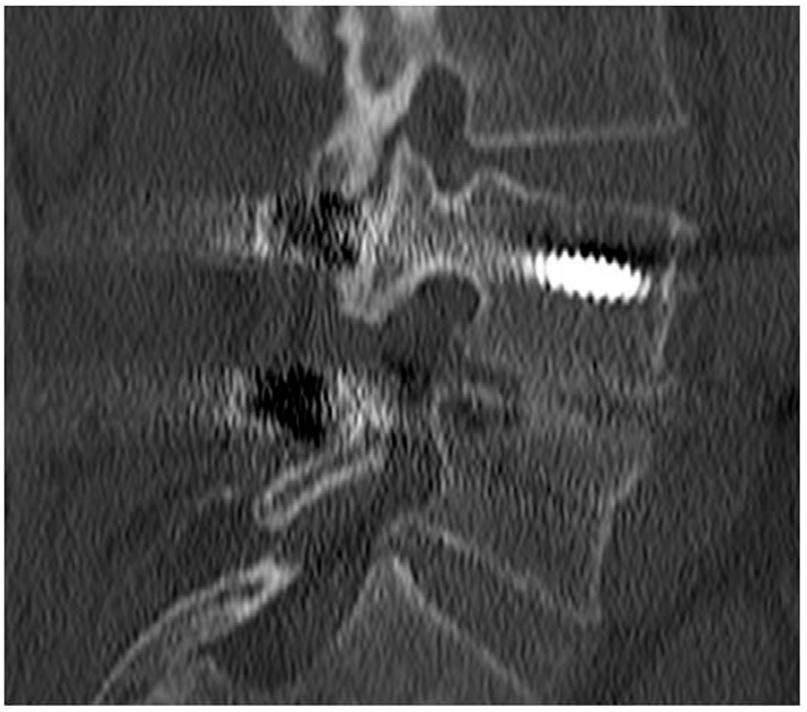
CT scan performed at 18-month follow-up, showing the absence of fusion (pseudoarthrosis) at levels L4–L5 and L5–S1 where SintLife was used.

Concerning the length of spinal fusion, nine patients (56%) had one fusion level, three patients (19%) had two fusion levels, and four patients (25%) had three fusion levels (Table 2). Three patients, all of them treated at two levels, were not evaluated at the follow-up because of inflammatory reactions occurring after surgery. Thus, spinal fusion was evaluated by an independent radiologist on CT scans performed at 12-month follow-up in 21 out of 27 treated levels. For each level, the fusion rate was assessed at the side where SintLife was used, and the results obtained according to Brantigan classification are reported in Table 3. The successful degree of fusion was about 62% (considering the levels with C, D, and E grades) (Table 3). The results represented by the Brantigan score were confirmed by quantification of the structural homogeneity of the bone graft area (ROI) (data not shown). Two cases of spinal arthrodesis with SintLife are reported in Figure 2 and Figure 3.

**TABLE 3:** Evaluation of the fusion status according to the Brantigan score in the presence of SintLife.

Brantigan score	Number of levels fused	%
A	2 levels	9.5
B	6 levels	28.6
C	3 levels	14.3
D	2 levels	9.5
E	8 levels	38.1

The first case was a 46-year-old female affected by degenerative disc disease L4–L5 and L5–S1 and treated with posterior stabilization L4–S1, PLIF L4–L5, and L5–S1. SintLife was apposed on the left side at L4–L5 and L5–S1 levels. At 18-month follow-up, a complete fusion has been detected at CT scan evaluation (grade E) (Figure 2), associated with significant improvement of clinical outcomes (VAS decreased from score 9 to score 1; ODI decreased from score 66 to score 31; and EQ-5D increased from 20 to 90).

The second case was a 61-year-old female affected by degenerative disc disease L4–L5 and treated with PLIF L4–L5. SintLife was apposed on the left side at the L4–L5 level. At 18-month follow-up, no fusion was detected (grade A) (Figure 3). However, a clinical outcome improvement was recorded (VAS decreased from score 5 to score 2; ODI decreased from score 30 to score 16; and EQ-5D increased from 60 to 80).

**FIGURE 4:**
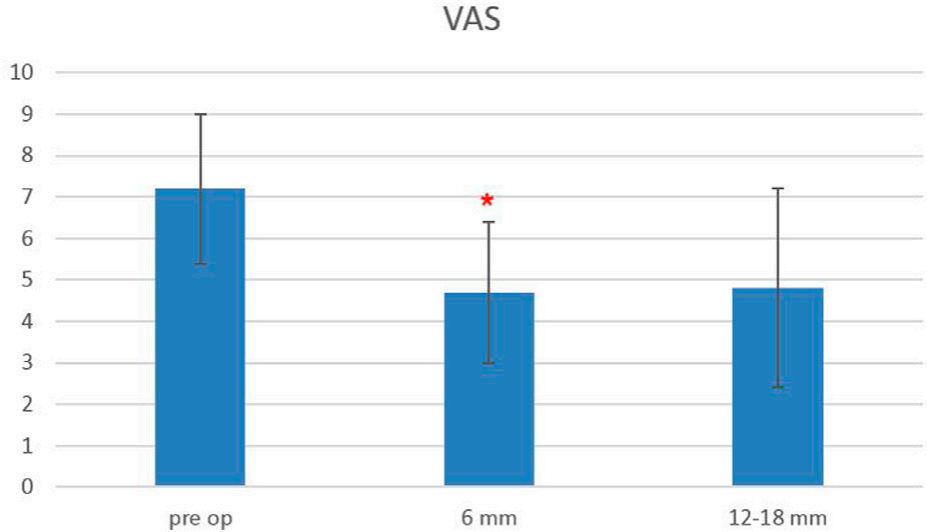
Plot of visual analog scale (VAS) scores evaluated pre-operatively, at 6- and 12–18-month follow-ups. The red asterisk depicts a significant difference between post-operative and pre-operative values.

Considering all the patients, the VAS score at baseline was 7.2 ± 1.8, and it decreased to 4.7 ± 1.69 at 6-month follow-up, while it remained stable at 12–18-month follow-up (4.8 ± 2.4), with a statistically significant difference between baseline and follow-up scores, starting from 6 months after surgery (*p* < 0.0004) (Figure 4).

**FIGURE 5:**
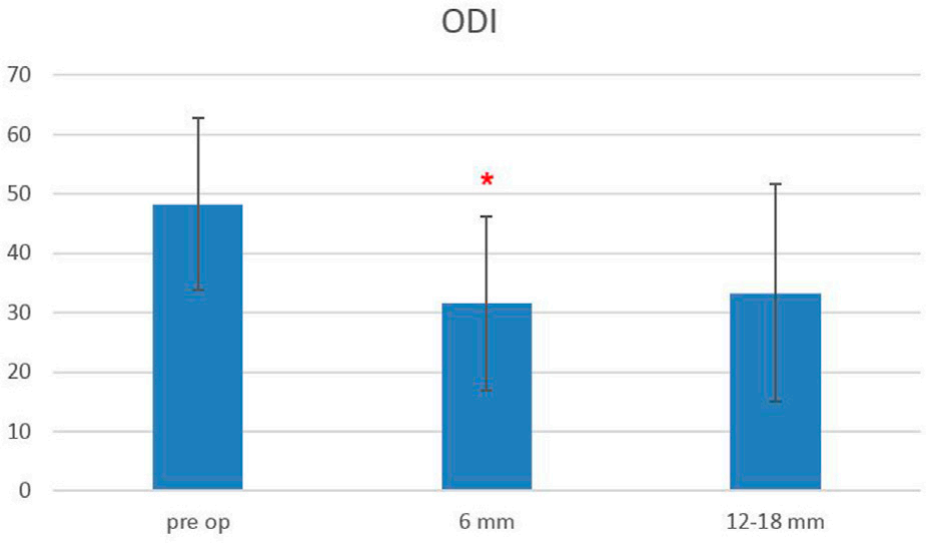
Oswestry Disability Index (ODI) scores evaluated pre-operatively, at 6-month and 12–18-month follow-ups. The red asterisk depicts a significant difference between post-operative and pre-operative values.

**FIGURE 6:**
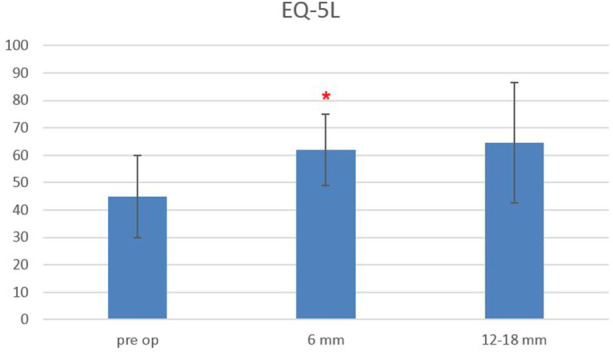
EuroQoL-5D (EQ-5D) scores evaluated pre-operatively, at 6-month and 12–18-month follow-ups. The red asterisk depicts a significant difference between post-operative and pre-operative values.

The Oswestry Disability Index at baseline was 48.3 ± 14.5, and it decreased to 31.6 ± 14.7 at 6-month follow-up and remained stable at 12–18-month follow-up (33.3 ± 18.3), with a statistically significant difference between baseline and follow-up scores, starting from 6 months after surgery (*p* < 0.0006) (Figure 5). The EQ-5L score at baseline was 45 ± 15, and it increased to 62 ± 13 at 6-month follow-up, and it was 64.5 ± 22 at 12–18-month follow-up, with a statistically significant difference between baseline and follow-up scores, starting from 6 months after surgery (*p* < 0.0003) (Figure 6).

Differences between ODI, VAS, and EQ-5D scores at 12–18-month follow-up, as compared to 6-month FU values, were not statistically significant, and a sensitive analysis performed by considering only those patients who underwent all three follow-up visits (*n* = 13) confirmed the trend.

Three adverse events (i.e., inflammatory reactions) were recorded in the follow-up period, with one requiring surgical debridement and the remaining treated with anti-inflammatory agents. According to the surgeon’s surgical notes, all the events were not classified as related or possibly related to the device. Two events were indeed rather related to inflammatory phenomena patients already suffered from. This was confirmed by elevated pre-operative values of C-reactive protein, as revealed by hematological investigations. The third event occurred after contamination of the surgical wound by *Enterococcus faecalis*, as ascertained by bacteriological tests.

No serious adverse events (i.e., immunological reactions, early/late infections, deep wound infection, implant mobilization, and early bone graft resorption) were recorded.

## Discussion

Ceramic-based bone graft substitutes (CaP, B-TCP, and hydroxyapatite) have been widely utilized since mid-1950s to reduce the need of iliac crest bone harvesting, given the complications associated with this procedure. As a family, ceramics can widely vary according to the different compositions, manufacturing, porosity, and structure.

Hydroxyapatite, a naturally occurring mineral form of calcium apatite with the formula Ca_5_(PO_4_)_3_(OH), has a stoichiometric formula (Ca/P ratio = 1.67) and a chemico-physical composition which are very similar to the mineralized phase of human bone. The porous structure of HA, with its macropore network and the micropore interconnection, induces rapid vascular and mesenchymal invasion, providing a specific cell flow and the optimal environment for cells to attach, proliferate, and finally differentiate into functional osteoblasts. HA shows high biomimetic properties, osteoconductive potential, and excellent biocompatibility. Through the extended surface-hydrated layer, linked to the crystal nanometer size, HA-based materials are able to exchange surface ions with the surrounding fluids, as needed to fulfill primary biological functions. Because of these properties, throughout years, HA-based bone grafts have evolved as “new generation” biomaterials able to “mimic” the osteoregenerative processes typically found in the human bone mineral turnover. This was performed by the addition of specific ionic species (cations or anions) which have been progressively introduced into the formulation of synthetic apatites. Magnesium is certainly one of the most important bivalent ions associated with biological apatite: it is one of the most abundant minerals in the human body, and approximately 50% of Mg^2+^ is naturally present in the composition of bone tissue. Mg^2+^ enables the HA crystal cell structure to become unstable and more biologically active, promoting rapid cell-mediated material resorption, promoting new bone formation, and remodeling by cross-talking with progenitor cells at the molecular level (Landi et al., 2008; Kurien et al., 2013; Barbanti Bròdano et al., 2014).

In the last few years, we had experience with an Mg^2+^-enriched HA biomaterial (SintLife) and tested its potentiality as a bone graft substitute in *in vitro* experiments and in *in vivo* animal models. Since 2006, Landi et al. (2006) demonstrated that Mg-substituted hydroxyapatites improved the behavior of mesenchymal stem cells in terms of adhesion, proliferation, and metabolic activation compared to stoichiometric HA.

More recently, *in vitro* experiments revealed an active interaction between SintLife and human mesenchymal stem cells (hMSCs), with an improvement of cell metabolic activities and bone remodeling (Barbanti Brodano et al., 2012; Manfrini et al., 2013). An *in vivo* study on sheep treated for postero-lateral fusion with SintLife or autologous bone showed the deposition of new bone tissue provided by SintLife, without qualitative and quantitative differences with respect to new bone formed with autologous bone graft (Barbanti Bròdano et al., 2014). Other studies (Pola et al., 2011; Sartori et al., 2014) demonstrated good osteointegration and deposition of new bone tissue using SintLife, thus confirming our previous findings.

With the present work, we took another step forward and investigated the use of SintLife in human spinal arthrodesis, with the aim to confirm the performance of the bone graft substitute.

The present prospective clinical study showed 63% of solid fusion by the use of an Mg-HA bone graft substitute (SintLife) alone for spinal arthrodesis.

To date, only few studies have investigated the fusion rate of CaP derivative biomaterials (i.e., HA, doped-HA, B-TCP, etc.) used alone, as compared to the gold standard autologous bone. Alimi et al. (2017) showed an overall fusion rate of 80% in degenerative disease patients treated with a silicate-substituted calcium phosphate (Si-CaP) ceramic bone graft in spinal fusion procedures.

At 6-month follow-up, Jenis and Banco (2010) showed 35% of fusion with silicate calcium phosphate, which increased to 76.2% and 76.5% at 12 and 24 months, respectively. Similar results (i.e., 90% of bony fusion after 12 months) were reported by Nagineni et al. (2012) in 2012.

In a review, Buser et al. (2016) showed an overall fusion rate of 70%–90% with the use of different hydroxyapatite-based bone grafts used alone, as compared to autograft. Other relevant literature reports high rates of fusion (of about 70–100%) obtained by the use of HA-based materials combined with autologous bone graft or autograft derivatives (BMA, ICBG, LAG, etc.) (Ransford et al., 1998; Delécrin et al., 2000; Thalgott et al., 2002; McConnell et al., 2003; Korovessis et al., 2005; Nickoli and Hsu, 2014).

Despite the reduced number of subjects enrolled in the study, results from this prospective study allow providing reliable considerations in terms of performance and safety of the device under analysis. SintLife has shown similar fusion rates, as compared to other CaP-derivative bone graft substitutes used alone.

Clinical parameters (ODI, VAS, and EQ-5L) used to measure the patient’s disability, pain, and quality of life assessed at 6 months and then at 12–18 months have shown a significant improvement. The improvement of clinical parameters at long follow-up periods is strictly linked to a solid fusion achieved, thereby confirming the effectiveness of the Mg-HA bone graft SintLife in achieving spinal fusions.

The safety profile of SintLife is confirmed by the lack of adverse events related to the material, as previously demonstrated by a post-marketing surveillance analysis (Barbanti Brodano et al., 2015) and confirmed in the present study. Of the three inflammatory conditions recorded in the previous literature reports, few cases of inflammatory reactions were reported following the use of bone graft substitutes for spinal fusion (Carragee et al., 2011; Kurien et al., 2013). These events are always related to previous patients’ clinical inflammatory conditions or hypersensitivity to some components of the device. In the present study, with the exception of one event related to a contamination by *Enterococcus faecalis*, two cases of imbalanced patients’ inflammatory conditions were identified following hematological analysis, which suggested no correlation between the event and the device, thereby confirming the safety of SintLife in procedures of spinal arthrodesis.

Limitations to the study are mainly related to the low number of subjects enrolled, moreover, conditioned by the worldwide COVID-19 pandemic which drastically reduced surgical activities during the period of the study conduction.

Taking into consideration that future research studies are needed to further investigate SintLife in spinal arthrodesis procedures, the biomaterial has shown a safe and effective profile.

## Conclusion

In conclusion, SintLife, a HA bone graft substitute enriched in magnesium ions, has shown a satisfying biocompatibility, a good performance in terms of spinal fusion and improvement of clinical outcomes, and a good safety profile and can represent a good alternative to autologous bone graft.

## Data Availability

The raw data supporting the conclusion of this article will be made available by the authors, without undue reservation.

## Abbreviations

BMA, bone marrow aspirate; BMPs, bone morphogenetic proteins; CaP, calcium phosphate; DBM, demineralized bone matrix; HA, hydroxyapatite; hMSCs, human mesenchymal stem cells; ICBG, iliac crest bone graft; LAG, local autograft bone; Mg, magnesium.
